# Contextual responses drive a unique laminar signature in human V1

**DOI:** 10.1016/j.isci.2025.112967

**Published:** 2025-06-19

**Authors:** Jurjen Heij, Luisa Raimondo, Jeroen C.W. Siero, Wietske van der Zwaag, Tomas Knapen, Serge O. Dumoulin

**Affiliations:** 1 Spinoza Centre for Neuroimaging, Amsterdam, the Netherlands; 2 Department of Computational Cognitive Neuroscience and Neuroimaging, Netherlands Institute for Neuroscience, Amsterdam, the Netherlands; 3 Department of Experimental and Applied Psychology, VU University, Amsterdam, the Netherlands; 4 Department of Radiology, Center for Image Sciences, University Medical Center Utrecht, Utrecht, the Netherlands; 5 Department of Experimental Psychology, Utrecht University, Utrecht, the Netherlands

**Keywords:** Natural sciences, Biological sciences, Neuroscience, Systems neuroscience

## Abstract

Neuronal populations in visual cortex integrate stimulus-driven input from the retina with contextual input from neighboring neurons, each targeting distinct cortical layers. Using line-scanning fMRI with precise targeting, we recorded depth-resolved responses in human visual cortex to stimuli tailored to each participant’s population receptive field (pRF) of the target patch. Stimuli in the center of the pRF evoked increasing responses toward the pial surface with a small peak at middle depths, consistent with feedforward input. Large stimuli in the surround elicited activity in superficial and deep layers, where descending connections terminate. Unexpectedly, medium-sized stimuli produced a complex pattern, possibly due to overlap from neuronal populations involved in stimulus- and context-related processes. Additionally, large surround stimuli evoked a negative deflection at middle depths, potentially reflecting suppression from lateral inhibitory circuits. These findings bridge invasive animal studies with human neuroimaging and highlight the potential for manipulating cortical computations non-invasively in cognitive neuroscience.

## Highlights

• Cortical layers may reveal stimulus-driven versus context-related input balance
• Line-scanning fMRI captured depth-resolved signals in living humans
• Stimulus-driven input increased toward pial surface with a peak in middle depths
• Contextual input targeted superficial and deep cortical depths

## Graphical abstract

**Figure:**
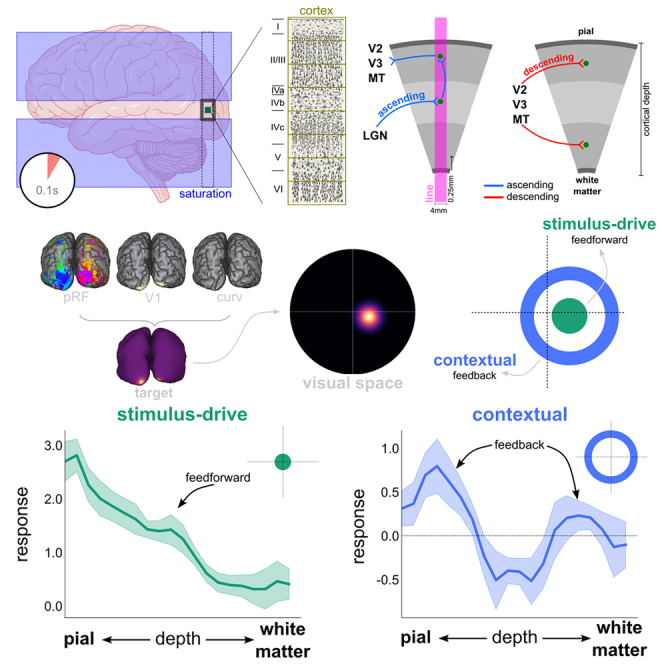

## Introduction

**Figure 1:**
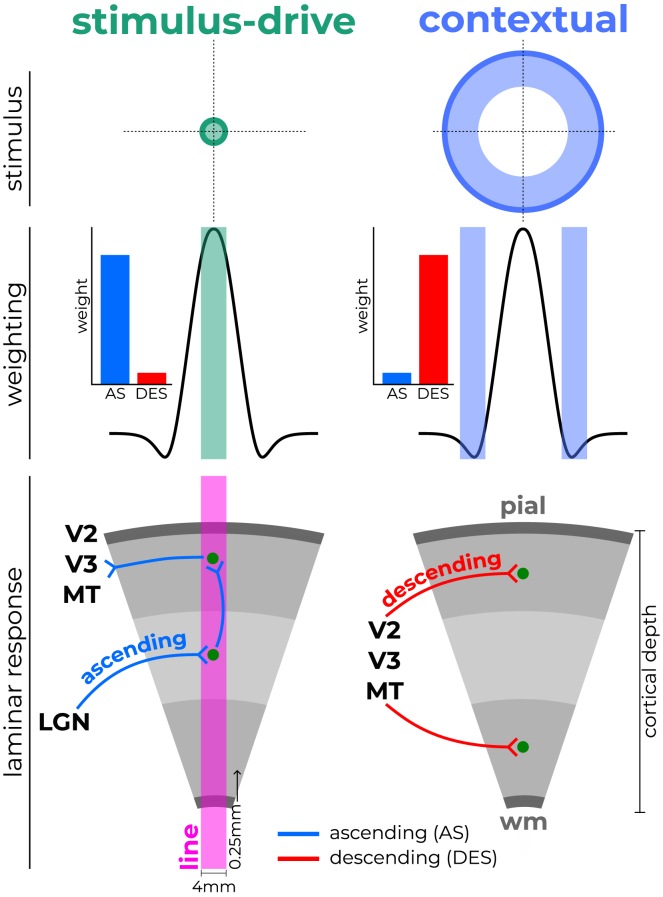
Laminar distribution of ascending and descending projections Stimuli falling within the classical receptive field (RF) primarily elicit stimulus-driven processes mediated by ascending (AS) connections (red). These signals are then propagated through connections spanning multiple cortical layers toward the surface and to neighboring regions. In contrast, stimuli on the flanks of the classical RF predominantly drive contextual processes, where information from neighboring areas is integrated via lateral and descending (DES) connections (blue) to the superficial and deep layers. Cortical layers thus provide a unique window into the interplay between stimulus-driven and context-related computations. Using line-scanning fMRI (purple rectangle), we can capture depth-resolved responses to custom-designed stimuli weighted differentially toward stimulus-drive or context with unprecedented detail.

Individual neurons in the early visual cortex respond to specific parts of the visual field, referred to as the receptive field (RF).^1^^,^^2^ The responses of these neurons are not solely reliant on stimulus-driven input; rather, they are complemented by context-related responses integrated from neighboring neurons^3^^,^^4^^,^^5^^,^^6^ (Figure 1, *top panels*). These responses can be modeled using surround-suppression or divisive normalization (DN). Specifically, DN has been shown to unify disparate responses including surround-suppression and compression.^6^^,^^7^ In DN, the responses of neurons are modeled as the ratio of the *activation* and *normalization pool*.^8^^,^^9^ The activation pool refers to the population of neurons predominantly driven by direct, feedforward input through ascending connections to the classical RF (blue arrows). The normalization pool consists of neurons that integrate contextual information from surrounding areas and contribute to balancing the responses of the activation pool via DN processes. While the activation pool primarily reflects direct input, the normalization pool mediates surround suppression and other forms of contextual modulation.^6^^,^^8^^,^^9^ From a biological perspective, contextual computations cannot be explained by ascending connections alone. Such computations require descending and lateral connections between neurons and neighboring areas.^4^^,^^5^^,^^10^^,^^11^^,^^12^^,^^13^

Ascending and descending connections terminate in specific layers of the cortex (Figure 1, *bottom panels*).^14^^,^^15^ Invasive neurophysiology studies in macaques have shown that stimuli within the classical RF elicit the earliest responses in layer 4 of primary visual cortex (V1).^4^^,^^10^^,^^16^^,^^17^^,^^18^^,^^19^ These neurons primarily receive excitatory input from ascending connections that originate in the lateral geniculate nucleus (blue projections).^2^^,^^17^^,^^20^^,^^21^ As stimuli extend beyond the classical RF, the visual system integrates contextual information from surrounding areas. These inputs are conveyed via descending (from neighboring neurons or areas to superficial and deeper layers of V1^4^^,^^5^; red projections) and lateral connections (present across the cortical column)^13^ corresponding to the normalization pool. Lateral connections primarily integrate information from nearby stimuli, contributing to near-surround modulation. In contrast, descending connections relay higher-order contextual information from distance regions, influencing far-surround processing.^3^^,^^22^^,^^23^ Therefore, altering stimuli such that they target the activation (classical RF) and normalization (surround) pool should engage different laminar connections (Figure 1, middle panels) and thus inform us how context-related computations are processed in the cortical layers of human V1.

We hypothesize that contextual modulation engages specific cortical layers, leading to distinct laminar profiles of BOLD activity to stimuli at the center of the RF compared to stimuli in the surround. While ultra-high field fMRI permits access to different layers of the cortex,^24^^,^^25^ conventional laminar fMRI acquisitions typically sample the cortex with 0.8mm isotropic resolutions.^24^^,^^25^^,^^26^^,^^27^^,^^28^^,^^29^ This yields about 2–3 data points across cortical depth, leading to increased signal mixing, especially in curved or vascular-rich areas, which could obscure laminar-specific modulations. To bypass these shortcomings, data from a region of interest (ROI) is typically upsampled,^26^^,^^30^^,^^31^ which requires extremely precise segmentation of the cortical sheet and accurate co-registration of anatomical and functional data.^32^ It also assumes that the depth-dependent fMRI signal across cortical depth (i.e., the tangential direction) remains consistent within the ROI^31^; blood vessels oriented 90° with respect to the B_0_-field can cause significant signal reduction.^33^^,^^34^^,^^35^ Given the highly complex folding pattern of the cortex,^36^ this results in high variability across the ROI. These issues translate to data with high statistical dependency and low variability across cortical depth and require averaging over a large ROI.^30^^,^^31^^,^^32^ Subpopulations of this ROI might exhibit variability in functional properties (e.g., RFs or center-surround configurations), reducing the specificity of the employed paradigm or acquisition scheme.^37^^,^^38^ To improve spatial and functional precision across cortical depth, acquisitions with higher resolutions are required. This can be achieved by using anisotropic voxel dimensions and/or reducing spatial coverage,^31^ while ensuring that the ROI is sampled at high resolution perpendicular to the cortical sheet.^39^^,^^40^^,^^41^

Initially developed in rodents,^42^ line-scanning fMRI has recently been adopted to investigate neuroscientific questions in humans.^41^^,^^43^^,^^44^ Line-scanning uses a single slice (2.5 mm thick) where the signal outside the region of interest is suppressed using 2 saturation pulses (4 mm gap)^42^^,^^43^ (Figure 1, purple beam). Thus, spatial coverage is sacrificed to sample responses from a specific patch of cortex with ultra-high spatial resolution (250 μm along cortical depth).^42^^,^^43^^,^^44^ While line-scanning fMRI does not necessarily offer higher SNR in absolute terms than conventional whole-brain acquisitions, it provides superior SNR per unit depth and higher spatial specificity perpendicular to the cortical sheet, which is critical for resolving laminar profiles. Specifically, line-scanning enhances the resolution of cortical depth imaging by providing 6–10 data points for a given cortical patch, whereas conventional laminar acquisitions provide 2–3 data points.^31^^,^^32^ Similar to animal neurophysiology, experiments can then be designed to target the functional properties of this cortical patch.^10^^,^^16^ Rather than measuring activity from single neurons, fMRI samples from large groups of neurons (20.000–30.000 neurons per cubic millimeter in cerebral cortex^45^^,^^46^). Similar to single neurons, these groups (or populations) respond preferentially to specific parts of the visual field as well,^47^^,^^48^ referred to as the population receptive field (pRF).^49^ Using our established selection-targeting method,^41^ we generated stimuli specifically tailored to the functional properties derived from computational modeling of participants’ targeted patches of the visual cortex. These stimuli targeted different populations of neurons facilitating stimulus-driven or contextual processes. Combining ultra-high spatial resolution with our functional targeting approach uniquely positions us to measure cortical depth-resolved responses and bridge human fMRI with non-human primate electrophysiology.

## Results

### Cortical-patch specific stimuli evoke differential responses

**Figure 2:**
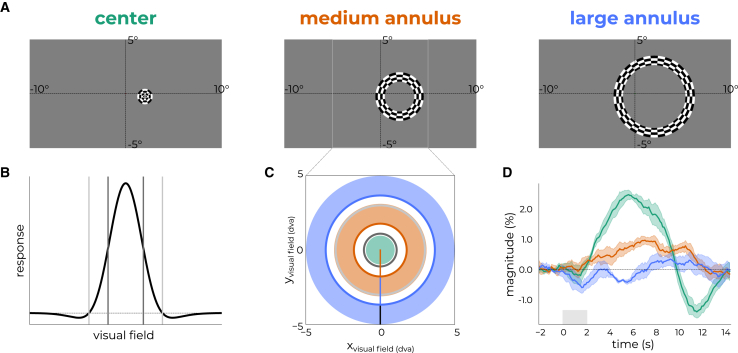
Participant-specific stimulus design procedure (A) Example stimuli as presented on the screen, targeting the location in visual space that is encoded by the target cortex patch. (B) Spatial response profile of target pRF with full-width at half-maximum (FWHM) denoted with dark gray bands and zero-crossings in light gray. (C) From the location of the target pRF (x, y) in visual space, we determined the smallest distance to the edge of the screen (x_visual field_ or y_visual field_) behind the MRI bore in order to present the largest stimulus possible without occlusion. The radius for the large stimulus (blue) was set to this distance (orange line + blue line + black line). The radius for the medium stimulus (orange) was set to be halfway of the distance between the center stimulus and large annulus. (D) Response profiles for the different stimuli averaged across the cortical depth for a representative participant (see Figure S2 for all participants). Data are represented as mean ± SEM.

Each participant was presented with a unique set of stimuli designed based on the functional organization of the target patch (see Figures 2A and S1). We deduced the locations of the zero-crossing and full-width half-max for each target pRF in visual space to highlight the stimulus configurations relative to its spatial profile (Figure 2B). We then derived size-tuning curves through simulations by passing stimuli of increasing sizes ranging from 0 to 10 degrees of visual angle (dva) to the estimates of the pRFs.^6^^,^^50^^,^^51^^,^^52^^,^^53^ Based on the size-tuning curve, we derived three stimuli centered on the pRF (Figure 2C) differentially eliciting stimulus-driven and contextual processes (Figure 1, *middle panels*). These stimuli indeed generated distinct responses collapsed over cortical depth, suggesting differential engagement of stimulus-driven and contextual processes (Figure 2D).

(1) *Center* stimulus (green): This stimulus consisted of a radial checkerboard stimulus with the size that elicited the maximum response according to the size-tuning curve (i.e., maximally targeting stimulus-driven processes);
(2) *large annulus* (blue): This stimulus consisted of a concentric ring subtending 2 dva, stimulating as much of the surround of the pRF as permitted by the physical dimensions of the screen. From the location of the target pRF in visual space, we drew the largest possible, non-occluded stimulus. The shortest distance served as the radius of the stimulus;
(3) *medium annulus* (orange): Similar to the large annulus, this stimulus consisted of a 2 dva-wide concentric ring with a radius halfway between the center and large annulus stimuli, preferably without spatial overlap with the other stimuli. All stimuli had 2 radial cycles per degree of stimulus size and 1 angular cycle per degree of stimulus size. In cases where parts of the screen were obstructed by the MR setup (e.g., transmit boxes, eye-tracker, MRI bore), we iteratively adjusted the stimuli based on verbal feedback from participants to ensure the stimuli were not occluded (Figure 2C).

To verify that responses localized to the intended target locations, we first averaged all the responses from data points across cortical depth and runs with identical timing (see method details: experimental setup). Response profiles were extracted from a time window starting 2 s prior to stimulus onset to 14 s after. This resulted in 2 (effective runs with different event timings) × 5 (events per run) profiles for each stimulus for each participant. The stimulus eliciting the largest response in the target pRF, according to the computational model, indeed resulted in the largest response of all stimuli (Figure S2). To formally test the response magnitude across events, we averaged the response over a period around the peak of the response to the *center* stimulus (Figure S2, insets) and entered this into a linear mixed-effects model (Table S1). The model demonstrated significant differences between stimulus types (*p* < 0.001). Using Tukey HSD pairwise comparisons, we found that the estimated mean response for the center stimulus (1.48 ± 0.094) was significantly higher than the response to the medium annulus (−1.22 ± 0.087; Δ = 1.22, 95% CI = 1.00–1.44, *p* < 0.001) and large annulus (−1.36 ± 0.087; Δ = 1.36, 95% CI = 1.14–1.58, *p* < 0.001). The difference in mean response between the medium and large annulus was not significant (Δ = −0.14, 95% CI = −0.36–0.07, *p* = 0.27). This confirms that the line-scanning approach was positioned in the cortical patch with the selected pRF.

### Context drives responses at superficial and deeper depths

The number of data points across the cortical depth of the target patch varied across participants due to differences in cortical thickness, ranging from 6 to 10 data points. To standardize the depth profiles for analysis, we re-gridded the data so that 20 evenly spaced points covered the cortical depth for all participants. Because of the increased resolution in the laminar direction, this study potentially has reduced interdependence across depth compared to previous studies that sampled 2–3 data points to 20 cortical depths.^26^^,^^30^^,^^31^^,^^54^^,^^55^^,^^56^ As we are not measuring individual layers of the cytoarchitecture, we discuss the results in terms of the three-compartment model (superficial, middle, and deep depths).^14^ Note that we use the three-compartment scheme only as a descriptive shorthand; our analysis does not force the data into exactly three levels, nor do we compare characteristics of the profiles of just three bins.

**Figure 3:**
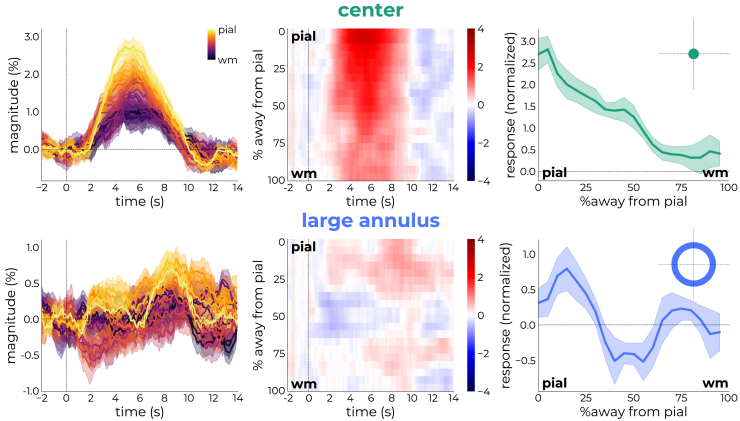
Response evolution for the center (green) and large annulus (blue) stimuli The left column represents the response profiles as extracted from the time courses. The middle column represents depth-by-time plots colored by magnitude. The right column is generated by taking the response profile to the center stimulus averaged across participants and cortical depth (hrf_template_) as template to generate cortical depth-dependent weights. This effectively collapsed the profiles over time, allowing more insights in the cortical profile. Using this weighting, the center stimulus elicited responses that increased toward the pial surface (pial) with a small peak at the middle depths. The large annulus elicited positive responses more reserved to superficial and deeper depths with negative responses at middle depths. These results are in line with electrophysiological and anatomical predictions about the laminar distribution of contextual integration. Data are represented as mean ± SEM∗1.96 (95% confidence intervals).

We then produced depth-by-time plots,^42^ colored by magnitude, to illustrate the evolution of responses across time and depth (Figure 3, left and middle columns). The center stimulus, designed to elicit stimulus-driven processes by virtue of being in the center of the pRF, resulted in a clear positive BOLD response across all cortical depths. In contrast, the response signature of the large annulus, designed to elicit contextual processes by virtue of being in the surround of the target pRF, was markedly different (Figure 3; bottom row). Most notably, the response seemed to be constrained to the superficial and deeper depths, while avoiding the middle depths for the majority of the time window (Figure 3; bottom row, middle panel). More specifically, and surprisingly, the middle depths even showed a deflection below zero relative to the other depths.

To visualize this effect more clearly, we collapsed the responses across depth over time using the group response to the center stimulus averaged across depth as a template. This approach is favored over the usage of a time window (as used in Figure S12) due to variability in response latency across participants. We calculated a weighted average to quantify how well the participants’ depth-dependent response profiles for other stimuli matches the profile for the group-averaged response to the center stimulus. This was achieved using the expression:

$$depth\;profile=\frac{\Sigma \left(hrf_{depth}\;\cdot \;hrf_{template}\right)}{\Sigma (hrf_{template)}}$$

where hrf_depth_ represents the cortical depth-dependent response profiles for the other stimuli, and hrf_template_ corresponds to the profile of the center stimulus (averaged over participants and cortical depth). The result provides a scaling factor that indicates how closely the depth-dependent responses of other stimuli align with the characteristic shape of the center stimulus profile (Figure S2, green profile in bottom-right panel labeled as “average”). A high value was assigned if the average profile was strongly represented in the profile of individual depths, and a low value if the opposite was true. To reduce noise, we normalized the profiles by subtracting each participant’s individual mean from the profiles and adding back the mean across participants.

For the response to the center stimulus, responses near the superficial surface (*pial*) were more strongly represented by the average profile compared to deeper depths (*wm*), a pattern often observed in the laminar fMRI literature.^34^^,^^41^^,^^57^^,^^58^^,^^59^^,^^60^^,^^61^^,^^62^ Additionally, a small peak can be observed in middle cortical depths (see also ref. ^35^), which could be putatively linked to the termination site of ascending projections.^14^^,^^15^ In contrast, the response profile to the large annulus exhibited peaks in response magnitude at the termination sites of descending projections^4^^,^^5^ (see Figure S3A for participant-specific profiles). This was not an artifact of voxel selection (Figures S3B and S12), normalization strategy (Figure S3C), weighting method (Figure S3D), or interpolation (Figure S4). This stimulus also produced a negative deflection in the middle depths. While this was not hypothesized *a priori*, this could potentially reflect suppression from lateral inhibitory circuits (see discussion for alternative explanations).

In addition to the primary echo combination approach (sum-of-squares), we repeated the analysis with a T_2_∗-weighted combination,^44^^,^^63^ which adapts weights voxel wise according to local T_2_∗ estimates. This method aims to balance BOLD sensitivity and signal stability while mitigating superficial vein bias. Results from this method yielded qualitatively similar laminar profiles, indicating robustness of our findings to the choice of combination strategy (Figure S5).

### Modeling the laminar profiles for large annuli

**Figure 4:**
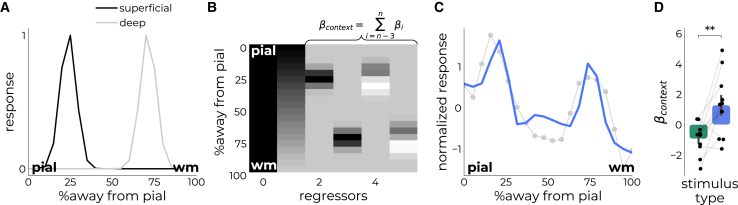
Modeling contextual integration across cortical depth for the large annulus (A) Based on invasive anatomical and functional findings, we expected strong responses in superficial (peak at 25%) and deep depths (peak at 75%) (red shaded boxes). We modeled these using a single Gaussian distribution for each termination site (gray profiles). We added derivative terms to each distribution to allow for participant variability in the exact location of the positive peaks. (B) From the full design matrix, we summed the beta values over the last 4 regressors representing the contextual component (*β*_context_). (C) Model fit of a representative participant’s response to the large annulus. (D) Comparison of summed beta-estimates from the contextual component (*β*_context_) of the model for center and large annulus stimuli. ∗∗*p* < 0.01. Data are represented as mean ± SEM.

To quantify this effect, we defined a descriptive model with two Gaussian distributions representing the peaks at superficial (peak at 25%) and deep (peak at 75%) cortical depths (Figures 4A and 4B). This model was based on (1) the observation that descending connections carrying context-related information from higher-order areas are received in superficial and deeper layers of V1,^14^^,^^64^ and (2) findings from an earlier animal electrophysiological experiment that used a strikingly similar setup in which these layers demonstrated responses to a large annulus.^16^ Derivative components were added to each Gaussian distribution to account for individual differences in the anatomy of the target patch, allowing the peaks to slightly shift (Figure 4B). To account for carry-over effects from deeper into superficial depths,^35^^,^^65^^,^^66^^,^^67^^,^^68^ we included a linear term due to the limited data points and ease-of-use.^34^ We then summed the beta values from the context-related model components (*β*_context_: double peak+derivatives, excluding the linear term, Figure 4B), which enabled us to quantify the extent to which a response reflected stimulus-driven or contextual processes (Figures 4C, 4D, and S6).

A one-way ANOVA revealed a significant main effect of stimulus type (*F*_1,20_ = 15.34, *p* < 0.001, partial η^2^ = 0.43), wherein the large annulus stimulus had higher beta values for the contextual component of the model (1.63 ± 0.61) compared to the center stimulus (−1.18 ± 0.38), *t*_10_ = 4.01, *p* = 0.002, Cohen’s *d* = 1.37. We evaluated the impact of draining vasculature in two ways. First, instead of using a linear component to account for the draining vein effect, we used a negative exponential component increasing toward the surface - better reflecting the macrovascular contribution (Figure S7). Second, we deconvolved the profiles across cortical depth using the vascular model from Markuerkiaga et al. ^65^^,^^69^ (Figure S8). Based on histological data^70^ and vascular modeling,^67^ they derived how each layer affects subsequent layers (see Table 1 in Marquardt et al.^69^ or Figure 3F in Markuerkiaga et al; ^65^). The deconvolved profiles were then entered in the same model as described above. Neither of these vascular correction methods altered the observed activation differences or conclusions. This suggests that stimuli eliciting predominantly contextual processes resulted in responses at sites where descending connections terminate, whereas stimuli eliciting stimulus-driven processes resulted in a strong BOLD response across cortical depth with a putative peak in middle depths where ascending projections terminate.

### Medium annulus produced mixed response

**Figure 5:**
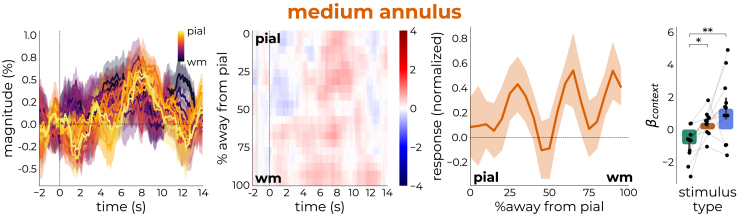
Medium annulus produces mixed response The first panel represents the response profiles as extracted from the time courses. The second panel represents depth-by-time plots colored by magnitude. The third panel represents the medium annulus response weighed by the response to the center stimulus. Whereas the large annulus elicited responses close to superficial depths (∼15% away from pial surface, Figure 3, bottom right panel), the medium annulus elicited responses in multiple sites across cortical depth (mean ± SEM∗1.96). The last panel represents the model outcome of all stimulus events Data are represented as mean ± SEM). The medium annulus fell in between the center stimulus and large annulus (center and large annulus stimuli taken from Figure 4D).

In the previous section, we examined responses to stimuli that were designed to maximally elicit either stimulus-driven (center) or contextual (large annulus) processes. Presenting a stimulus that lies somewhere in between these extremes should result in a response pattern that is intermediate (medium annulus). Indeed, the response profile deviated from the other two stimuli. The time courses are noisier and the profile across depth less specific (Figure 5, first and second panel). This could be due to vascular carry-over effects obscuring true responses (Figure S8) or because the stimulus configuration differed across participants (Figure S1; Figure S9); for some participants, the medium annulus may have hit stimulus-driven populations, whereas for others it was fully in the surround, driving contextual processes. The response profile of the medium annulus showed widespread activation with peaks closer to middle depths compared to the large annulus (Figure 5, third panel), potentially reflecting lateral processing.

Even though the mixed nature of the stimulus renders interpretation difficult, we subjected the laminar profiles elicited by the medium annulus to the same model described earlier (Figures 5 and S10). A one-way ANOVA across all stimulus types revealed a significant main effect of stimulus type on the beta values from the context-related model components (*F*_2,30_ = 7.11, *p* = 0.003, partial *η*^2^ = 0.32). Post-hoc analysis with Holm’s correction (Figure 5, last panel) showed that the context-related component was significantly higher for the medium annulus (0.75 ± 0.38) compared to the center stimulus (−1.18 ± 0.38; *t*_10_ = 3.15, *p* = 0.03, Cohen’s *d* = 1.51). The difference between the large and medium annulus was not significant (*p* = 0.22). Together, these results show that stimuli designed to elicit contextual processes result in responses that differ from stimuli eliciting stimulus-driven processes through ascending connections.

## Discussion

### Probing contextual responses with line-scanning fMRI

In this work, we used DN to probe the laminar signature of stimulus-driven and contextual processes. DN is observed across the brain in multiple systems^8^^,^^9^^,^^71^^,^^72^^,^^73^ and is therefore often considered a canonical computational operation that the brain employs for various purposes.^9^ Central to DN is the principle that the output of a given neuron depends not only on its direct stimulation (activation) component but also on the integration of signals from nearby neurons (normalization component). These processes provide insights into the nature of feedforward and feedback mechanisms: the activation component is driven by ascending bottom-up connections terminating in the middle layers of the cortex,^2^^,^^17^^,^^20^^,^^21^ while contextual integration from neighboring neurons or areas arises from descending connections terminating in the superficial and deeper layers of the cortex.^4^^,^^5^^,^^14^^,^^15^ Thus, altering the relative contribution of stimulus-drive and context can reveal processing circuits within the layers of the cortex. In this study, we applied our selection and targeting framework to investigate these responses in humans using ultra-high-resolution line-scanning fMRI.^41^ Specifically, we targeted a patch of cortex with defined functional properties and presented stimuli designed through computational modeling to probe the laminar signatures of stimulus-driven or contextual processing. Our results demonstrate that cortical depth-dependent responses varied based on the preference to elicit stimulus-driven or contextual processes.

### Distinct context-dependent laminar signature

Stimuli designed to maximally elicit stimulus-driven processes (*center*) resulted in responses across the cortical depth with a putative peak in the middle depths, whereas contextual stimuli (*large annulus*) elicited positive responses in the superficial and deeper cortical depths and a negative response in middle depth (Figure 3). Many studies have attempted to separate feedforward from feedback processing, but direct comparison is rendered complicated due to different experimental paradigms. Such paradigms include texture-segmentations,^10^ low-spatial frequency stimuli,^18^ high-contrast drifting gratings,^74^ and line segments.^19^ Differences in experimental setup will target different neuronal populations across cortical depth, often resulting in differences in the laminar profile of spiking.^75^

Yet, Bijanzadeh et al.^16^ performed a study with many similarities to the experimental setup presented in this work: In an attempt to study surround suppression, a special category of DN, they recorded responses across cortical depth to a similar set of stimuli: a stimulus inside the classical RF, an annulus stimulating the near surround, and an annulus stimulating the far surround. Similar to our work, they found that responses to stimuli in the surround were constrained to superficial and deeper layers of the cortex; layers where descending connections carrying context-related information from neighboring regions terminate.^14^^,^^15^ They also observed the earliest activation in the middle layers in response to a stimulus in the center of the RF. In the current study, we found a small peak in the middle layers as well, which became clearer after accounting for the carry-over effects (see next section).^35^ One important distinction is, however, that Bijanzadeh et al.^16^ mainly reported differences in latencies between stimulus-driven and contextual processes, whereas the current study reports on amplitude across cortical depth. The speed with which these signals are transmitted makes it complicated to capture using BOLD fMRI,^10^^,^^18^^,^^75^ even with fast acquisitions.^25^^,^^42^^,^^76^^,^^77^ Nevertheless, the similarities in experimental design and outcome (pattern of activation across cortical depth) highlight the possibility to link animal electrophysiological experiments with non-invasive human fMRI experiments.

While the observed effects align with the idea of DN, alternative explanations may also account for, or contribute to, the observed responses. Similar to the studies previously discussed,^3^^,^^5^^,^^16^ the results could be interpreted in many context-dependent processes such as lateral inhibition, surround-suppression, or differences in temporal dynamics between the stimuli. For example, the large annulus might engage lateral inhibitory circuits that suppress activity in the middle layers, while enhancing processing in superficial and deep layers.^4^^,^^10^^,^^78^ Alternatively, the large annulus might recruit distinct populations of excitatory and inhibitory neurons across layers, with inhibition dominating in the middle layers and excitation driving responses in superficial and deep layers.^79^^,^^80^ The large annulus might also elicit non-linear response patterns, where neurons in the middle layer are less responsive due to saturation or competitive suppression.^5^^,^^9^ From a vascular perspective, the BOLD signal in superficial and deep layers may be amplified due to their proximity to larger veins and the influence of descending inputs. These factors, combined with potential suppression of middle-layer activity (layer 4) by the large annulus, could account for the observed depth-specific BOLD response patterns.^55^^,^^81^^,^^82^^,^^83^ However, different frameworks are unified by the specific circuits that underlie them: ascending, descending, and horizontal projections. Complementary approaches perturbing these processes may inform us further about how the cortex resolves them using the same circuit architecture.^75^^,^^84^

### Depth-dependent BOLD: Metabolic and vascular biases

The BOLD effect arises from a change in relative amounts of oxygenated and deoxygenated blood. It is therefore an indirect measure of neuronal activity, linked to particular neuronal activity such as local field potentials (LFP),^85^^,^^86^^,^^87^^,^^88^^,^^89^ or action potentials (in case of pRFs).^90^ Upon neuronal activation, metabolic demands trigger extra delivery of blood toward the site of activation.^91^ Deoxygenated blood, which distorts the magnetic field due to unpaired iron atoms, is pushed away, resulting in increased signal.^92^^,^^93^ Within the cortex, penetrating arteries branch off the pial network to supply the parenchyma of nutrients,^68^^,^^83^ while veins drain the deoxygenated paramagnetic blood unidirectionally toward the pial surface.^94^^,^^95^^,^^96^ The corollary of this process is 2-fold: 1) BOLD signal changes are larger at superficial cortical depths where the deoxygenated blood is pooled^97^^,^^98^^,^^99^^,^^100^^,^^101^^,^^102^ and 2) signals from deeper depths influence the signal at superficial depths, which has been referred to as the draining vein effect, carry-over effects, or leakage problem.^35^^,^^65^^,^^66^^,^^67^^,^^68^ Strategies have since been developed to mitigate the effect of large pial veins, including acquisition,^103^^,^^104^^,^^105^^,^^106^ analysis,^35^^,^^65^^,^^97^^,^^107^^,^^108^^,^^109^^,^^110^ and experimental design^49^^,^^111^^,^^112^ (for further details, see these laminar fMRI reviews^26^^,^^30^^,^^32^^,^^75^^,^^84^^,^^113^^,^^114^^,^^115^^,^^116^).

Why, then, is the carry-over effect less visible for contextual stimuli? The center stimulus is designed to stimulate the center of the pRF, maximally eliciting the stimulus-driven processes. Such processes drive ascending inputs into the middle layers of V1^10^^,^^14^^,^^15^^,^^16^. The transmission of these inputs involves dense excitatory synapses and high-frequency spiking activity to propagate sensory input.^89^^,^^91^^,^^117^ The metabolically demanding processes increase local oxygen consumption and blood flow, producing a robust BOLD response.^89^^,^^91^^,^^118^ In contrast, the large annulus primarily engages lateral and descending inputs facilitating contextual processes.^119^ Contextual processes are modulatory, rather than driving (i.e., modulate existing activity instead of generating new action potentials), which requires less energy.^4^^,^^5^^,^^120^^,^^121^^,^^122^^,^^123^ This renders contextual processes metabolically less demanding compared to ascending inputs.^120^^,^^122^^,^^124^^,^^125^ The difference in metabolic demand between these inputs might explain the presence of the draining vein effect in the stimulus-driven condition but not the contextual condition as well as overall magnitude differences.

### Limitations of the study

The effective resolution of line-scanning is influenced by several factors, including the curvature of the targeted cortical sheet,^126^^,^^127^^,^^128^ participant motion,^129^^,^^130^ the quality of saturation slabs,^43^ targeting success,^41^ and positioning relative to the surface coils used for MR signal detection.^131^^,^^132^ The significant reduction in the field-of-view during line-scanning imposes limitations on its applicability for examining larger-scale processes, such as between-area communication. Although our multi-echo sum-of-squares combination increases sensitivity to BOLD contrast, it may also increase signal sensitivity to large surface vessels. As a result, superficial vein contributions may contaminate our laminar profiles and bias responses toward the cortical surface. We carefully selected our cortical patch to be tissue-dominated by excluding obvious veins using the mean functional image as vein mask during the selection procedure.^41^^,^^100^ Nevertheless, we tested the importance of the signal combination approach by comparing the SoS reconstruction to a T_2_∗-weighted combination which accounts for voxel wise decay and BOLD sensitivity. We observed similar laminar profiles using this alternative combination method.^44^^,^^63^ This consistency suggests that our main findings are not driven by the choice of echo combination method, though future studies could employ more advanced methods.

This setup is further complicated by the use of circular, flickering checkerboard stimuli designed based on pRFs estimated using a bar-sweep configuration,^24^^,^^49^ which were acquired using a different sequence,^24^ on different days,^133^^,^^134^^,^^135^^,^^136^ and with varying levels of thermal noise.^44^^,^^137^^,^^138^ These factors collectively affect the neuronal population (and therefore the pRF) that is ultimately targeted.^24^^,^^134^^,^^139^ Additionally, we modeled the responses using the DN-model, but given the focus on the surround in early visual cortex, these could have been modeled using the difference-of-gaussian model as well.^140^ Regardless of model choice, the pRF stimulus with bar configurations is primarily a spatial design that does not account for the time dimension. In the current setup, stimuli perturb neuronal populations for much shorter durations (2 s) compared to the bar configuration (15–20 s). It remains unclear how such changes in the temporal characteristics of stimuli influence processing dynamics across cortical depth.

Last, future work could develop more advanced definitions of contextual processing. This work operationalized this by using a descriptive biphasic model representing termination sites of descending projections. While this model is relatively simple and allows for some degree of interpretation, it does not provide a mechanistic account.

### Bridging neurophysiology and fMRI

In this study, we applied the selection and targeting framework of line-scanning to investigate contextual processing across cortical depth. This strategy mimics invasive electrophysiological setups in which a known target is probed across depth with electrodes. Using existing pRF data, we designed stimuli tailored uniquely for each participant to maximally elicit stimulus-driven and contextual processes. The advantages of this approach are 2-fold: (1) cortical depth is sampled by significantly more data points (6–10 vs. 2–3), reducing partial volume effects and minimizing the effects of large veins; and (2) a specific patch of cortex can be targeted, improving the specificity of the experimental paradigm. We demonstrated that the stimulus eliciting stimulus-driven processes (center stimulus) resulted in strong responses across all cortical depths, with particularly strong responses near the cortical surface and a small peak in the middle depths—the site where ascending connections from LGN terminate. In contrast, stimuli eliciting contextual processes produced responses more constrained to superficial and deeper depths—sites where descending context-related connections from neighboring areas terminate. These findings align with evidence from animal studies and computational models, highlighting the potential to establish links between animal methodologies and human research. The non-invasive nature of this experimental setup further offers new opportunities to explore cognitive manipulations in humans.

## Resource availability

### Lead contact

Requests for further information and resources should be directed to and will be fulfilled by the lead contact, Jurjen Heij (j.heij@herseninstituut.knaw.nl).

### Materials availability

This study did not generate new unique agents.

### Data and code availability

• Data in BIDS-format^141^ will be made available on request in compliance with GDPR regulations.
• The code for this paper is available in the following repositories: Preprocessing of fMRI, anatomical pipeline, and handling of line-scanning data: https://github.com/gjheij/linescanning; Line-scanning experiment: https://github.com/gjheij/LineExps/tree/main/ActNorm3; Analysis: https://github.com/spinoza-centre/holeresponse.
• Any additional information required to reanalyze the data reported in this paper is available from the lead contact upon request.

## Acknowledgments

This work was supported by a 10.13039/501100001722Royal Netherlands Academy of Arts and Sciences (KNAW) grant (2018, to S.O.D., W.v.d.Z., J.C.W.S., and T.K.), a Netherlands Organization for Scientific Research (10.13039/501100003246NWO) Vidi Grant (TTW VI. Vidi.198.016 to W.v.d.Z.), an 10.13039/501100003246NWO Vici grant (016. Vici.185.050 to S.O.D.). The Spinoza Center is a joint initiative of the KNAW—10.13039/501100020251Netherlands Institute for Neuroscience, Vrije University Amsterdam, Amsterdam University Medical Centra—locations AMC and VUmc.

## Author contributions

Conceptualization, T.K. and S.O.D.; methodology, J.H., T.K., and S.O.D.; investigation, J.H.; writing – original draft, J.H.; writing – review and editing, J.H., L.R., J.C.W.S., W.v.d.Z., T.K., and S.O.D.; funding acquisition, J.C.W.S., W.v.d.Z., T.K., and S.O.D.; resources, W.v.d.Z., T.K., and S.O.D.

## Declaration of interests

The authors declare no competing interests.

## STAR★Methods

### Key resources table

**Table:** 

REAGENT or RESOURCE	SOURCE	IDENTIFIER
**Software and algorithms**		

Pycortex	Gao, et al. (2015)	https://gallantlab.org/pycortex/
FreeSurfer	Fischl, 2012	https://surfer.nmr.mgh.harvard.edu/fswiki
MATLAB	MathWorks	https://www.mathworks.com/
Psychopy	Pierce, 2007	https://www.psychopy.org/

### Experimental model and study participant details

13 participants (ages 23–50 years, 5 female) participated in this study. All participants had normal or corrected-to normal visual acuity, were screened prior to the experiments to ensure MR compatibility, and provided written informed consent as approved by the ethics committee of the Vrije Universiteit Amsterdam. Some participants were scanned twice targeting a different pRF, resulting in a total of 18 individually sampled cortical patches.

### Method details

#### Experimental setup

The visual stimuli were generated using the Psychopy package,^142^ wrapped in exptools2 https://github.com/gjheij/exptools2. Stimuli were displayed on an MRI-compatible screen located outside the bore (Cambridge Research Systems 32″ LCD widescreen, 1920 × 1080 resolution, 120Hz refresh rate) and viewed by participants through front-silvered mirrors (example stimuli shown in Figure 2A). Each stimulus presentation consisted of an 8Hz flickering stimulus displayed for 2 s. The experiment started with a blank screen of mean luminance for the duration of the dummy scan (∼42s) and baseline (30s) before the stimuli started to appear. The inter-stimulus intervals (ISIs) were jittered following a negative exponential decay to reduce collinearity between subsequent events^143^^,^^144^^,^^145^ and were spaced far apart to enable epoching strategies. ISI_min_/ISI_max_/ISI_mean_ values were 14s/24s/18s, resulting in five stimulus presentations per stimulus per run (7 min). To maximize signal-to-noise ratio (SNR) while limiting predictability, we simulated two sets of stimulus presentation orders and intervals. These two variations were randomly presented to participants during the session. To ensure engagement, a small fixation dot was presented in the center of the stimulus, which changed color (red to green) at intervals following a negative exponential decay (ISI_min_/ISI_max_/ISI_mean_ = 4s/8s/6s). Eye movements were monitored using an EyeLink 1000 eye-tracker system at 1000Hz (https://www.sr-research.com), and participants were instructed to report color changes via a button press.

#### Vertex selection

Similar to our previous approach,^41^ vertex selection was performed using surface processing procedures from pycortex.^146^ We aimed to identify a vertex within the primary visual cortex (manually delineated based on polar angle maps) that met the following criteria: located in the eccentricity band subtending 1.5–3 dva, at least 1 dva away from the vertical meridian, with sufficient variance explained (r^2^ > 0.55) and reasonable pRF sizes (σ_1_ > 0.50 dva). A binary mask representing the surviving vertices was visually inspected using FreeSurfer’s FreeView. From this mask, we selected a vertex within a blob that was favorably positioned with respect to curvature and neighboring vertices. This approach ensured that responses from similarly behaving pRFs would be accurately projected into the line.

#### Data acquisition

The workflow includes two separate scan sessions typically acquired on different days. The first session is dedicated to the acquisition of anatomical information and whole-brain population receptive field (pRF) estimation (see ref. ^41^ for acquisition and experimental paradigm). In the second session, we perform our functional line-scanning experiment, targeting a specific location on the cortical surface. All acquisitions were performed on a Philips Achieva 7T MRI system.

The line-scanning functional acquisition used a modified multi-echo 2D gradient-echo sequence where the phase-encoding gradients are removed and two OVS bands are used to suppress signals outside the line.^43^^,^^44^ With this sequence, 94.3 ± 1.3% of undesired signals outside the region of interest is suppressed.^43^^,^^44^ A gap of 4 mm between the two OVS bands was used, resulting in a nominal resolution for the line of 4 × 2.5 × 0.25 mm^3^, with 0.25 mm in the laminar direction. Other parameters were: TR/TE_1-5_ = 105 ms/6 ms, 14 ms, 22 ms, 30 ms, 38 ms, readout bandwidth = 131.4 Hz/pixel, FA = 16°.^44^ Data were acquired using two custom-built high-density 16-channel surface coil arrays (total 32 channels) for signal reception^131^^,^^132^ and the NOVA coil for transmission (Nova Medical, Wilmington, MA). The gradient coil has a maximum amplitude of 40 mT/m and a 200 T/m/s maximum slew rate.

For registration, a 4-min whole-brain T_1_-weighted scan was acquired using the two-channel transmit coil to receive (Nova Medical, Wilmington, MA), at a resolution of 1.5 mm isotropic (FOV = 245 × 245 × 184 mm^3^ matrix = 164 × 163×184, TR/TE = 6.2 ms/3 ms, FA_1_/FA_2_ = 5°/7°, TR_MP2RAGE_/TI_1_/TI_2_ = 5500 ms/800 ms/2700 ms). Two short additional scans accompanied the line-scanning acquisition: for the nominal line representation, a slice image with phase encoding but without OVS bands was acquired. For line coil sensitivity maps used in reconstructing line-scanning data, a slice image with phase encoding and OVS bands was acquired.

#### Data reconstruction and quality assessment

The reconstruction of the line-scanning data was performed offline using MATLAB Gyrotools. Multichannel coil data were combined using a temporal signal-to-noise ratio (tSNR) and coil sensitivity-weighted sum-of-squares (SoS) scheme per echo.^43^^,^^44^ Multi-echo data were subsequently combined using a sum-of-squares operation to maximize signal stability and contrast-to-noise (CNR).^44^ To minimize habituation effects, stimuli were presented in two different orders. High-frequency noise was addressed by applying a Savitzky-Golay filter^147^ (31 samples, 3^rd^ order) before averaging runs with the same stimulus order.

Line-scanning fMRI is particularly sensitive to movement due to its limited coverage. To mitigate this, our participant pool consisted of highly experienced individuals, and movement was further restricted by securing the chin to the transmit coil with tape. The target area of interest was manually delineated on a run-to-run basis by identifying the CSF/gray matter and gray/white matter boundaries using the anatomical reference slice. For each event, we extracted and averaged the time period from 2 s before stimulus onset to 14 s after (Figures S11A and S11C). This process resulted in an average response to each stimulus for each participant across the different stimulus orders (Figure S11B). Based on the responses across the entire line, we confirmed that the stimuli evoked the most specific responses in the target area (Figure S11D).

Cortical locations were included if they met two criteria (Figure S12). First, the response to the center stimulus had to be larger than the responses to the other stimuli. Second, this stimulus had to exhibit the draining vein effect across cortical depth. To verify responsiveness, we used a general linear model (GLM) with a canonical HRF, where the prediction based on center stimulus events was entered as a regressor. This analysis yielded variance explained across the line, allowing us to confirm that the largest responses occurred near the intended target location. For depth profiles, we estimated the magnitude evolution within a 5–7 s window after stimulus onset. Since cortical depth was covered by a varying number of data points across participants, the data were regridded so that cortical depth was uniformly covered by 20 data points.^26^^,^^54^^,^^55^^,^^56^

In cases where discrepancies occurred between the selected data points and the variance explained, an additional shift was applied to voxel selection. For some participants, this adjustment resulted in cleaner responses to the center stimulus and an improved draining vein profile (black profiles). From the 18 individually sampled pRF responses, 11 (defined as *n*) were included in the final analysis. It is important to note that this procedure enhanced the precision of the results by increasing statistical power (higher *n*), but the findings remained consistent even without this adjustment (Figure S3B).

### Quantification and statistical analysis

Significance testing was performed using the Python packages *Pingouin*^148^ and *statsmodels*.^149^ For each comparison, tests for normality and homogeneity of variance were conducted, and the appropriate statistical test (parametric or non-parametric) was selected based on the results. For within-participant comparisons of stimulus responses, paired-samples t-tests were applied. Group comparisons were conducted using ANOVA, followed by Holm-corrected post-hoc tests when applicable. The significance level was set to α = 0.05. Unless otherwise specified, 95% confidence interval (SEM∗1.96) was used to quantify precision. The specific statistical tests, significance levels, and precision measurements are reported in the text and/or figure captions.
