# Supplementary material for: Contextual responses drive a unique laminar signature in human V1
Source: iScience. 2025 Jun 19;28(7):112967. doi: 10.1016/j.isci.2025.112967 (PMC12270667; doi:10.1016/j.isci.2025.112967)
Supplement: Document S1. Figures S1–S12 and Table S1 [file mmc1.pdf]

iScience, Volume *28*

Supplemental information

Contextual responses drive a unique laminar signature in human V1

Jurjen Heij, Luisa Raimondo, Jeroen C.W. Siero, Wietske van der Zwaag, Tomas Knapen, and Serge O. Dumoulin

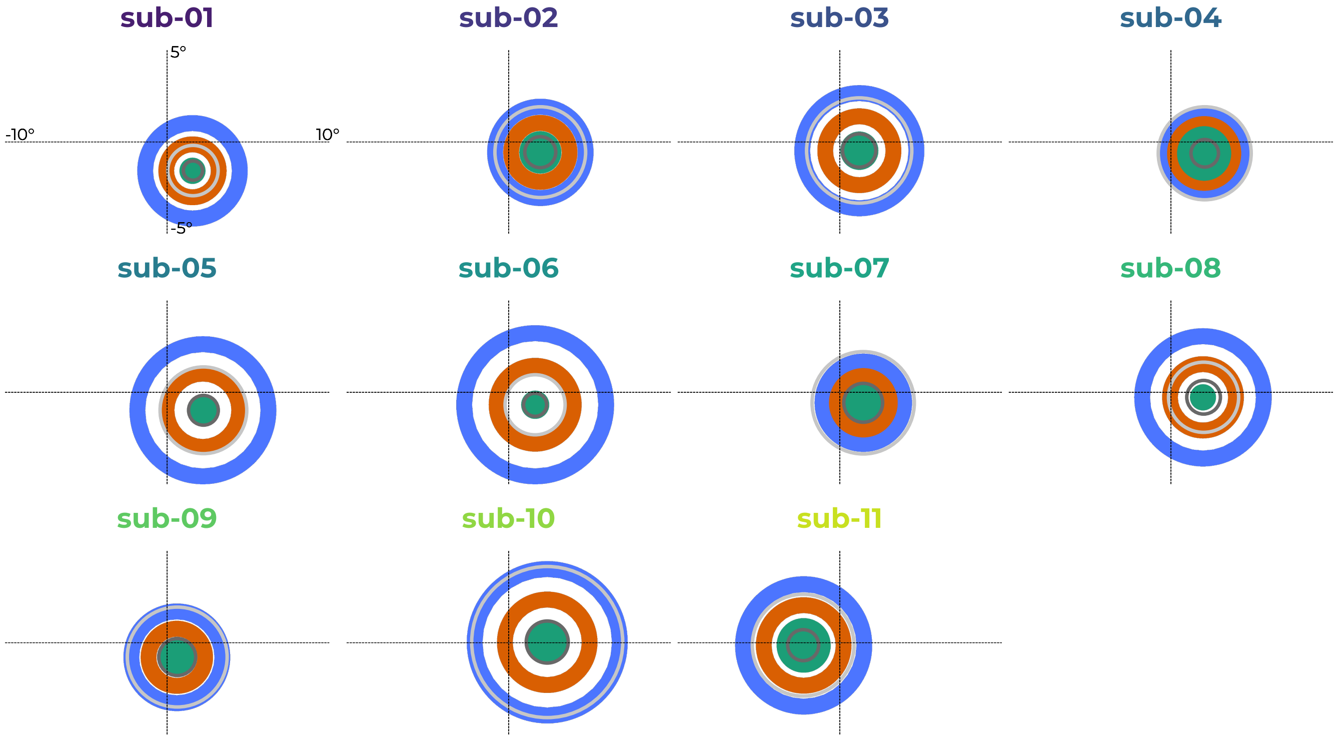

Figure S1: Participant-specific stimulus configurations as derived from computational modeling. The dark gray circle indicates the location of the full-width half-max of the spatial profile, whereas the light gray circle indicates the zero-crossing (Figure 2B). Most participants had the target pRF in the right hemifield which is encoded in the left hemisphere. This figure is related to Figure 2.

Table S1: Mixed-linear model regression results. We averaged the response over a time period around the peak of the response to the *center* stimulus (*intercept*) and entered this into a linear mixed-effects model. The medium annulus was defined as *suppr_1* and the large annulus as *suppr_2*. Post-hoc Tukey HSD pairwise comparisons further showed that response magnitude was significantly lower for the medium and large annulus compared to the center stimulus, while no significant difference was observed between the two annulus stimuli. These results highlight the effect of stimulus type on response magnitude, suggesting robust and consistent reductions in response during stimuli biased towards the normalization pool, with minimal variability across participants. The small residual variance (Scale=0.4405) and moderate random intercept variance (Group Var=0.059±0.05) further support the robustness of these results.

Model:	MixedLM	Dependent Variable:	gm
No. Observations:	330	Method:	REML
No. Groups:	11	Scale:	0.4405
Min. group size:	30	Log-Likelihood:	-345.0529
Max. group size:	30	Converged:	Yes
Mean group size:	30.0		

	Coef.	Std.Err.	z	P> |z|	[0.025	0.975]
Intercept	1.473	0.097	15.243	0.000	1.284	1.663
event_type[T.suppr_1]	-1.275	0.089	-14.251	0.000	-1.451	-1.100
event_type[T.suppr_2]	-1.424	0.089	-15.917	0.000	-1.600	-1.249
Group Var	0.059	0.050				

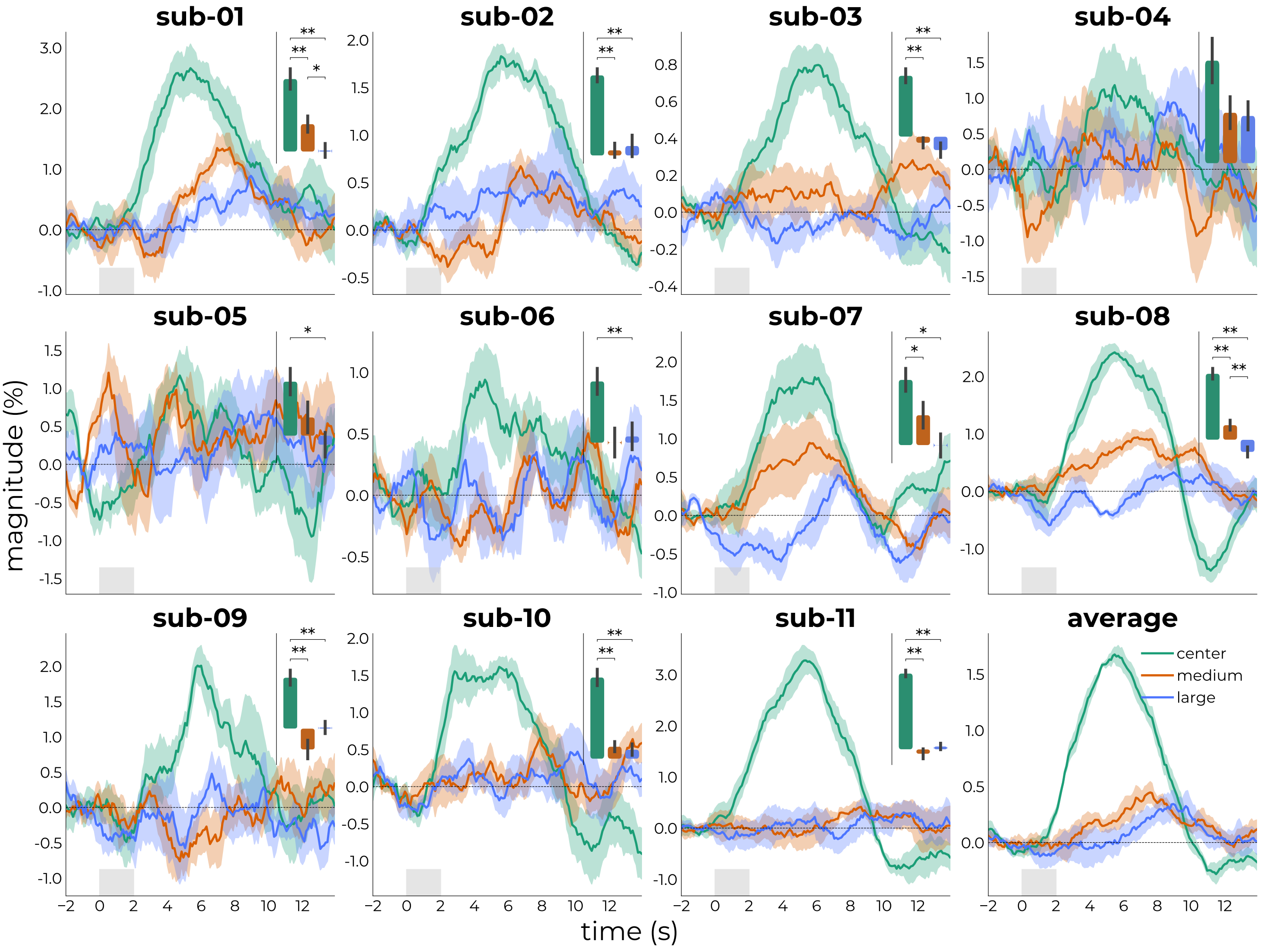

Figure S2: Participant-specific responses averaged across the cortical voxels to individually tailored stimuli. The bottom right panel represents the average across all participants. Insets represent participants-wise values extracted from the time point where the response to the *center*-stimulus peaked. Individual Friedman ANOVA's (as the data from some participants violated the normality assumption) were performed to formally test differences in response magnitude across stimulus types. Significance (asterisks) was based on post-hoc analyses using Wilcoxon signed-rank tests with Holm's correction. In all participants, the optimal stimulus (eliciting the largest response given the parameters of the pRF at the target location) according to the computational model resulted in the largest fMRI response in all participants (and reached significance in all but one participant). This figure is related to Figure 2D. *$p<0.05$, **$p<0.01$.

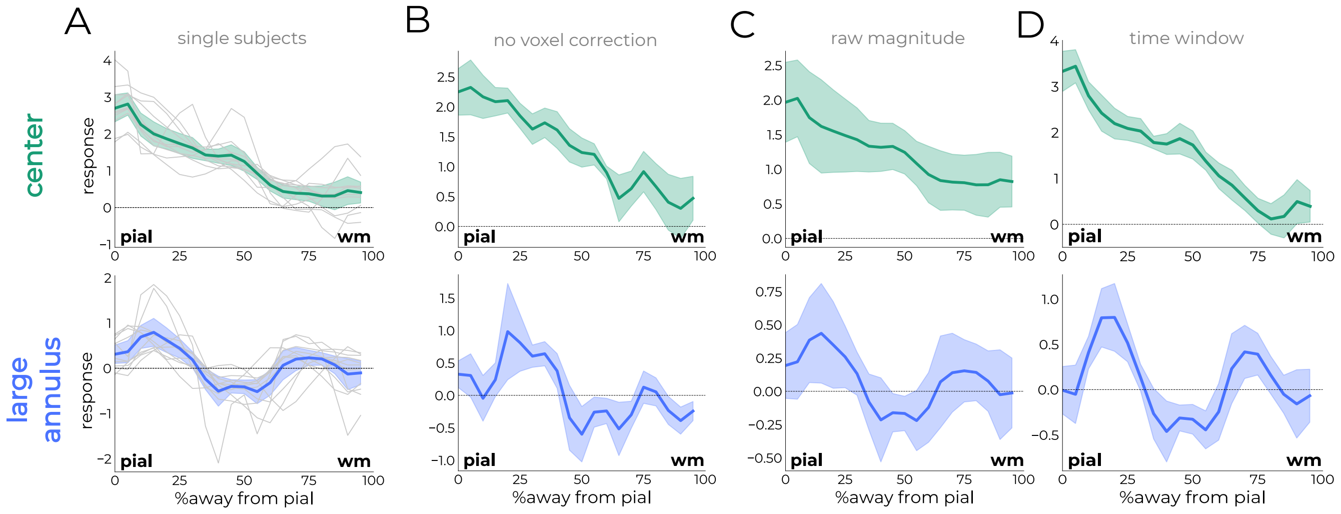

Figure S3: Control analyses. (A) Laminar results as shown in Figure 3, including participant-specific profiles. Although some variability between participants is observed, there is a remarkable consistency is localization of two peaks that align with termination sites of descending projection carrying feedback signals predicted from anatomical and electrophysiological work. (B) Response profiles of the dataset of included participants had the voxel correction approach not been applied (see also Figure S12). This led to a reduced number of participants, but the result remained clear. (C) Response profiles resulting from subject-specific magnitude responses (no normalization approach). (D) Response profiles resulting from a time window approach, rather than using the response to the center stimulus as template. This approach uses a window between 5-7s (similar to Figure S12) to extract magnitude values. However, subjects have idiosyncratic response latencies, rendering such an approach hard to generalize. This figure is related to Figure 3.

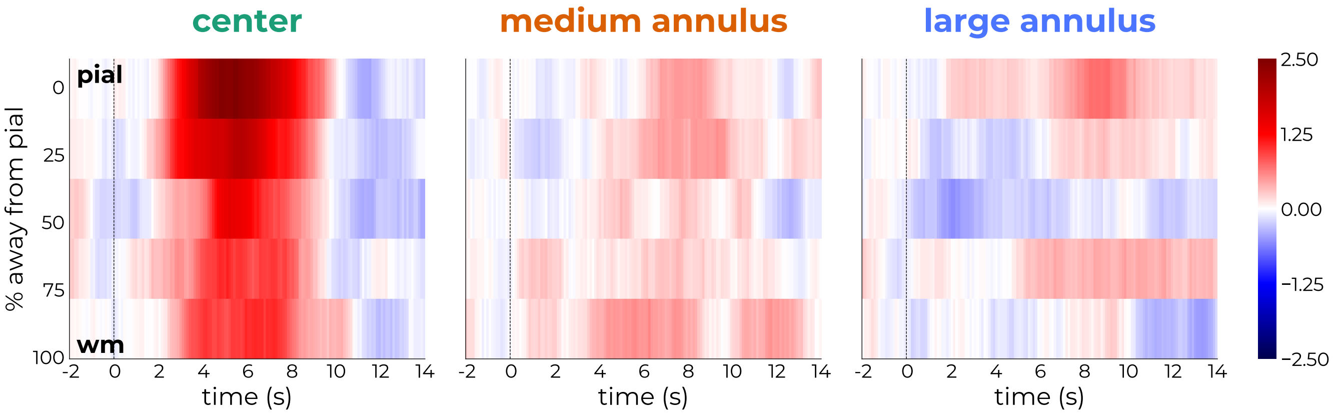

Figure S4: Laminar results without interpolation (related to Figure 4). Each participant's cortical column of interest was covered by 6–10 data points (voxels). In contrast to contemporary laminar studies that rely on interpolating 2–3 voxels to 20 depths (see refs[1–4]), line-scanning permits sampling many more data points across cortical depth. To synchronize the number of depths across participants, line-scanning uniquely allows averaging multiple voxels into a single compartment. In other words, the large number of data points allows for downsampling (averaging across multiple data points), rather than upsampling (expanding few data points into many). This boosts SNR without relying on interpolation. Using this approach, we could still observe the preference for signal processing in upper and deeper layers for stimuli targeting more of the normalization pool. The draining vein effect for center stimuli was also preserved. This figure is related to Figure 3.

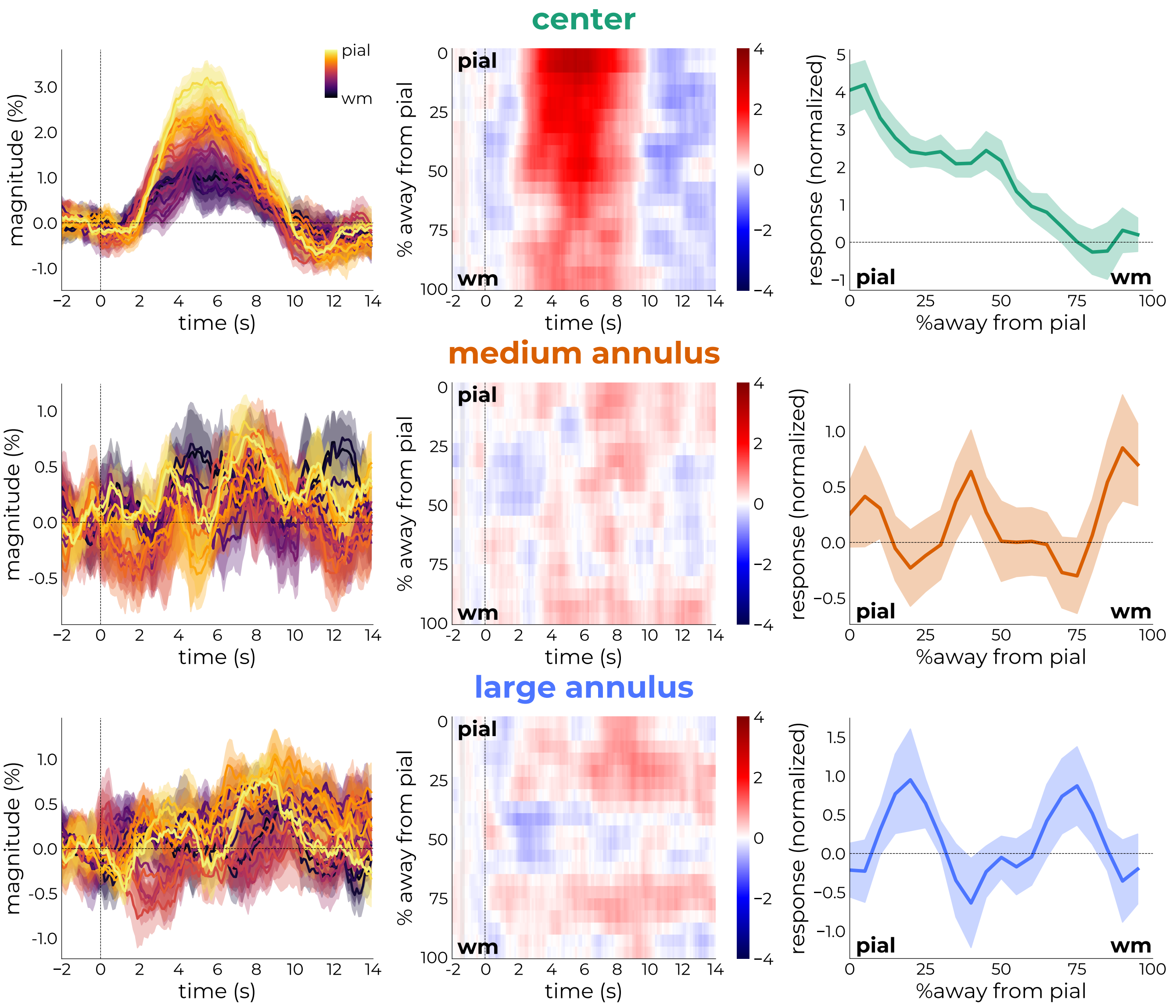

Figure S5: Laminar profiles from T_2*-weighted echo combination. Laminar time courses and normalized profiles for center, medium annulus, and large annulus stimuli using T_2*-based echo combination[5,6]. Results closely match those obtained using sum-of-squares, supporting the robustness of our findings.

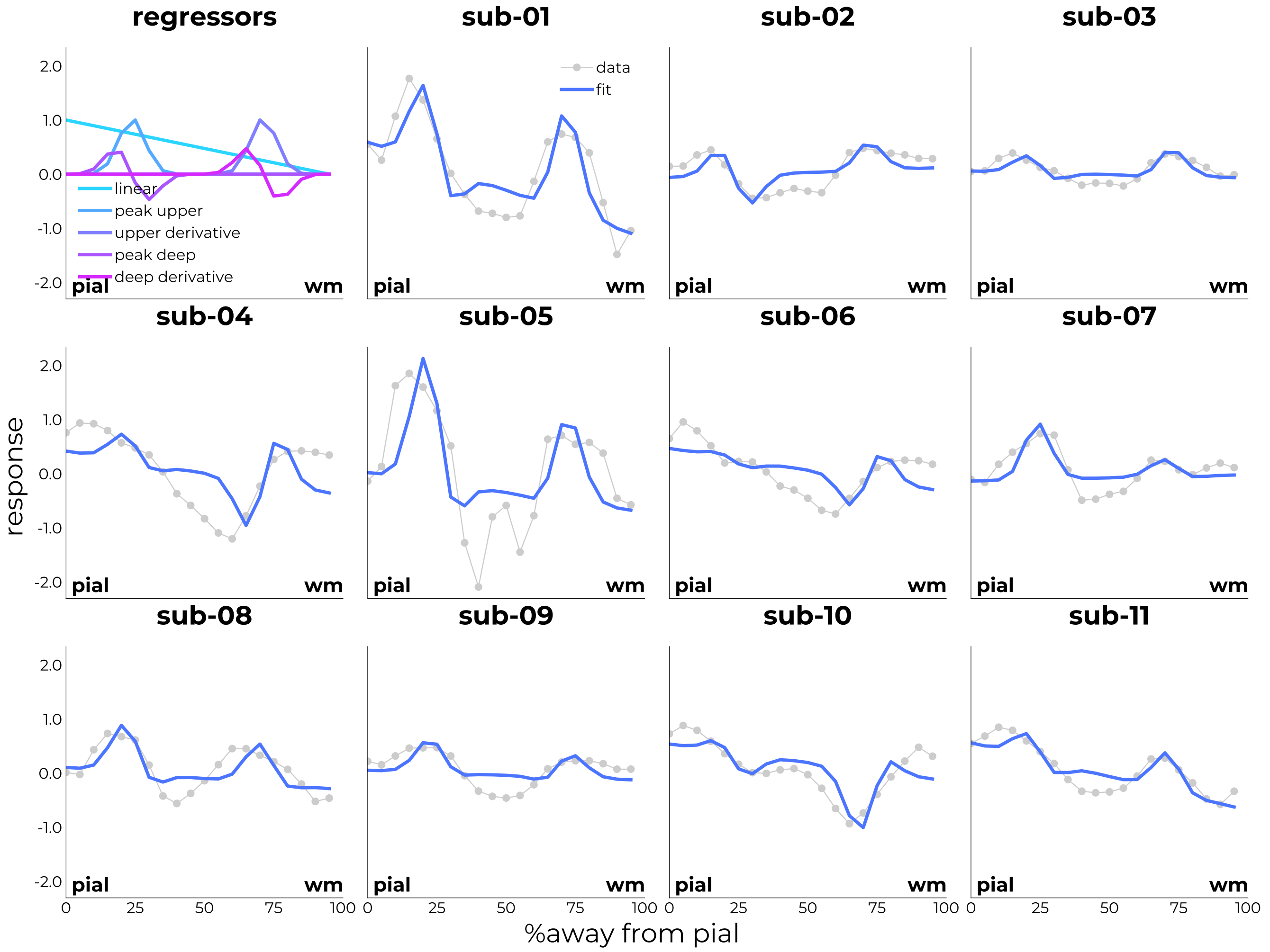

Figure S6: Participant-specific results of fitting the contextual model to cortical depth-dependent responses to the large annulus stimulus. The top-left panel represents the regressors entered into the model: a linear trend and two regressors and their derivatives (to allow shifts in exact location of the peaks) representing the termination sites of descending projections based on anatomical and functional studies. This figure is related to Figure 4.

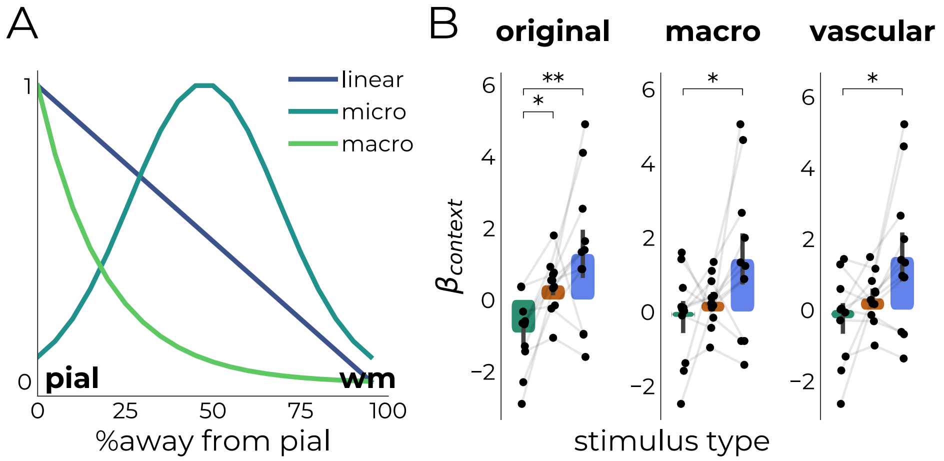

Figure S7: Other methods for vascular correction. (A) Instead of using a linear trend to account for carry-over effects, we evaluated the model outcome using regressors for microvasculature (Gaussian distribution in the middle depths, as microvascular density is highest in these layers[7–9]) and macrovasculature (negative exponential decaying from superficial layers down to the deep layers typically observed in laminar BOLD fMRI[10–17]). (B) Compared to the original model (first panel), including macrovascular components (macro) or both micro-/and macrovascular components (vascular) did not affect the results between the center stimulus and large annulus. **$p<0.01$, *$p<0.05$. This figure is related to Figure 4 and 5.

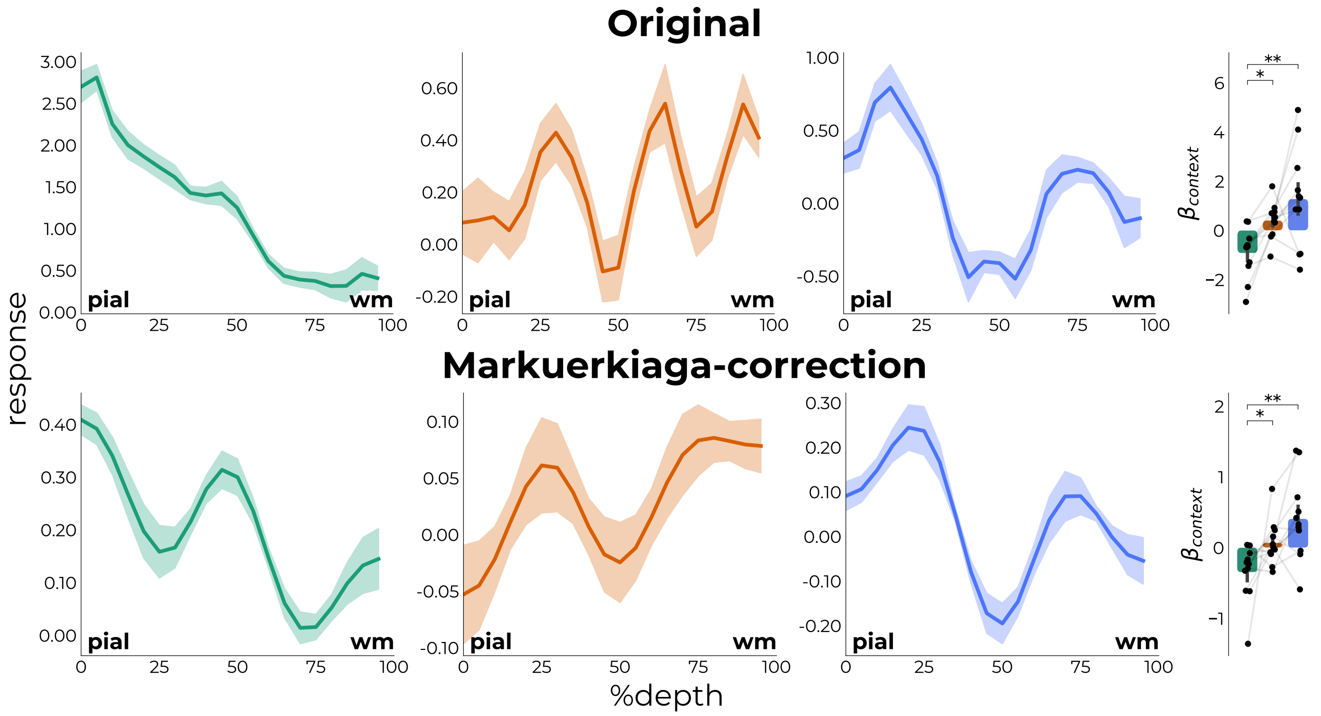

Figure S8: Markuerkiaga and colleagues developed a method to account for the carry-over effect often observed in laminar BOLD fMRI[18,19]. This method is a spatial deconvolution method, where the signal of each subsequent layer going from deep to superficial layers is weighed based on histological data[20] and a vascular model[21] (see Table 1. in ref[19] and Figure 3F in ref[18]). Because these weightings assumed a 5×5 structure, we upsampled the design to 20×20 to match the weightings to our depth-dependent data using Lanczos interpolation. This matrix was then applied using an OLS (ordinary least-squares) approach with ridge regression to correct the profiles for the carry-over effects. The top row shows the profiles as presented in Figure 3 and 5 in the main text, with the β_{context} over different stimuli (same bar plot as the last panel in Figure 5 of main text). After correction for carry-over effects, the center stimulus exhibited a pronounced peak in the middle depths. This could be indicative of feedforward processing, as these projections from LGN terminate in these layers[22,23]. The response to the medium annulus showed a less variable profile compared to the original profile, suggesting that some of these responses may have been mediated by vascular effects. The response to the large annulus, however, did not show marked differences with the original profile. This is indicative that the vascular organization impacted this response to a lesser extent. We applied the same contextual model (see Figure 4 in main text) to the correct profiles. This showed that the pattern remained the same, suggesting that vascular effects did not drive the reported results. This figure is related to Figure 3, 4, and 5.

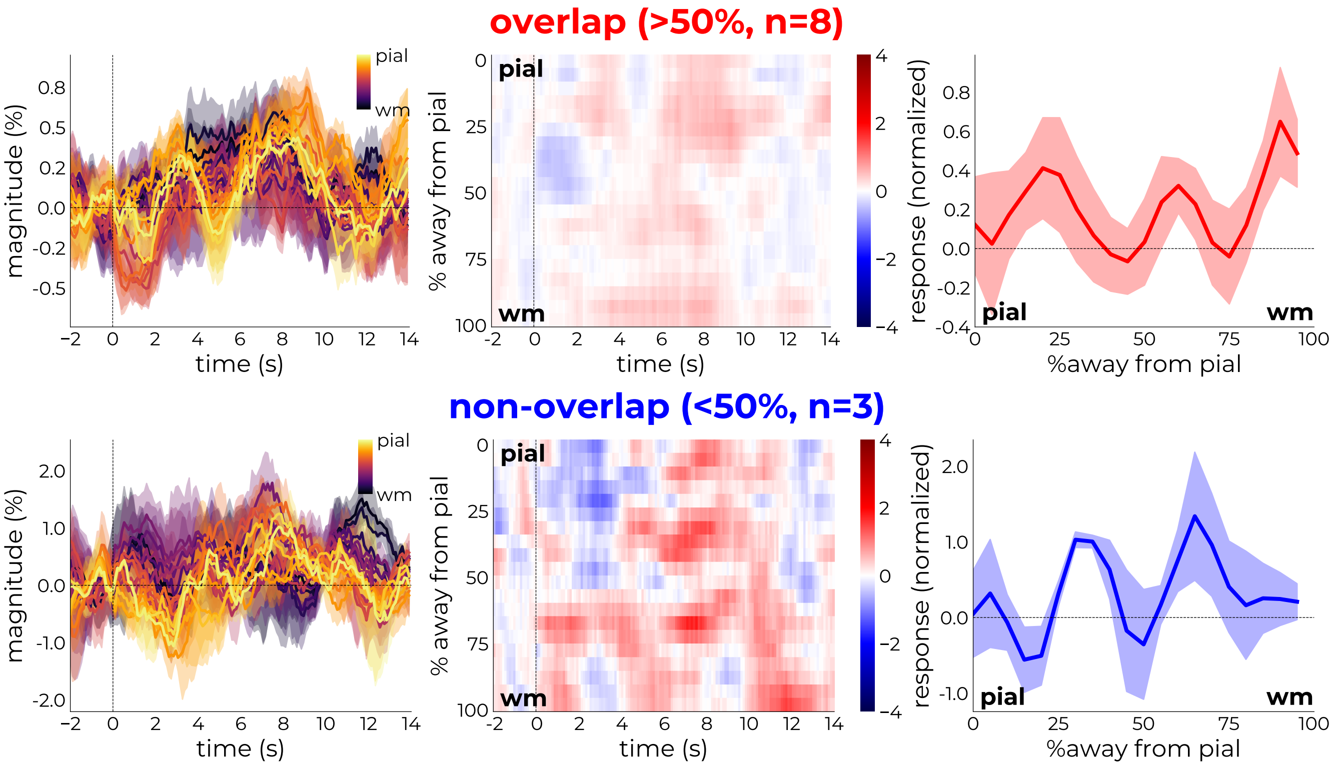

Figure S9: Using the 1D profile of the target pRF and stimulus configuration of participants (see Figure S1), we estimated the percentage of stimulus pixels residing outside the zero-crossings. The more pixels outside the zero-crossing, the more the stimulus can be considered to target the "near" surround[24]. Although separating the already limited sample into rather unequal groups (3 non-overlap, 8 overlap), the non-overlap group showed a cleaner response compared to the mixed response in Figure 5. Here, response peaks could be observed in cortical layers reminiscent of the near-surround response in Bijanzadeh, et al.[24]. These results clean up the responses to the medium annulus somewhat and highlight the intricate relationship between computational modeling and experimental design. This figure is related to Figure 5.

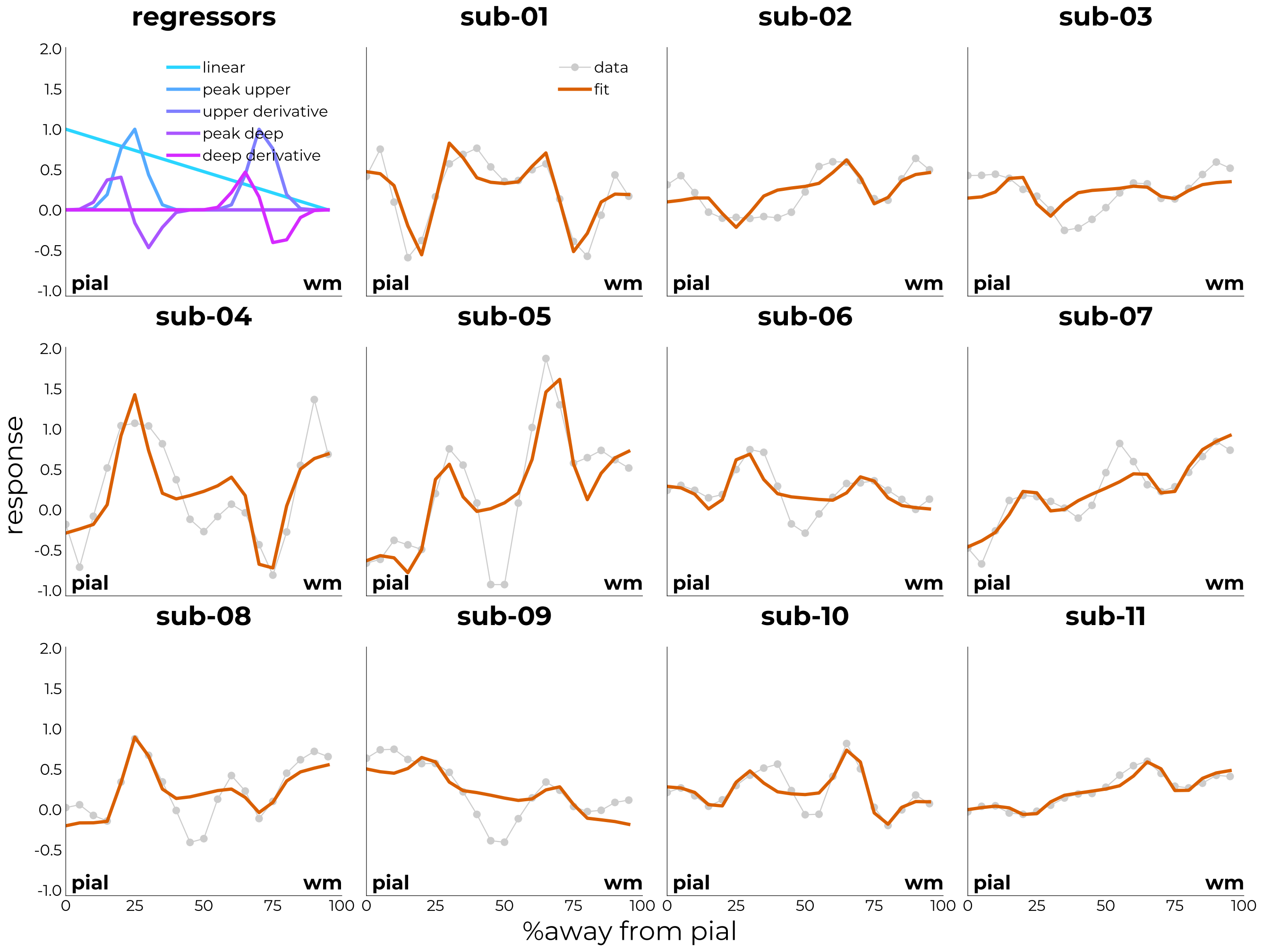

Figure S10: Participant-specific results of fitting the contextual model to cortical depth-dependent responses to the medium annulus stimulus. The same regressors as Figure S6 were used. This figure is related to Figure 5.

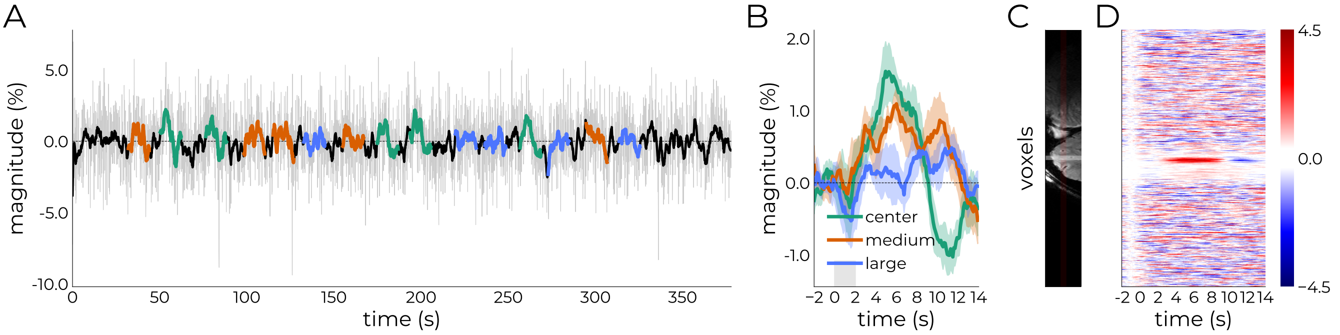

Figure S11: Epoching strategy and targeting success. (A) Example raw (light gray) and low-passed time course (black) or a single run with intervals around each respective stimulus onsets highlighted in their corresponding color (green = center, orange = medium annulus, and blue = large annulus). (B) Response profiles derived from averaging the epochs from all runs (shaded area represents ±SEM over the five epochs per event). (C) Section of anatomical reference slight with the gray shading representing the selected cortical ribbon. (D) Response evolution across the entire line, showing response preferences in the target location. This figure is related to the section “*Quantification and statistical analysis*” of the STAR Methods.

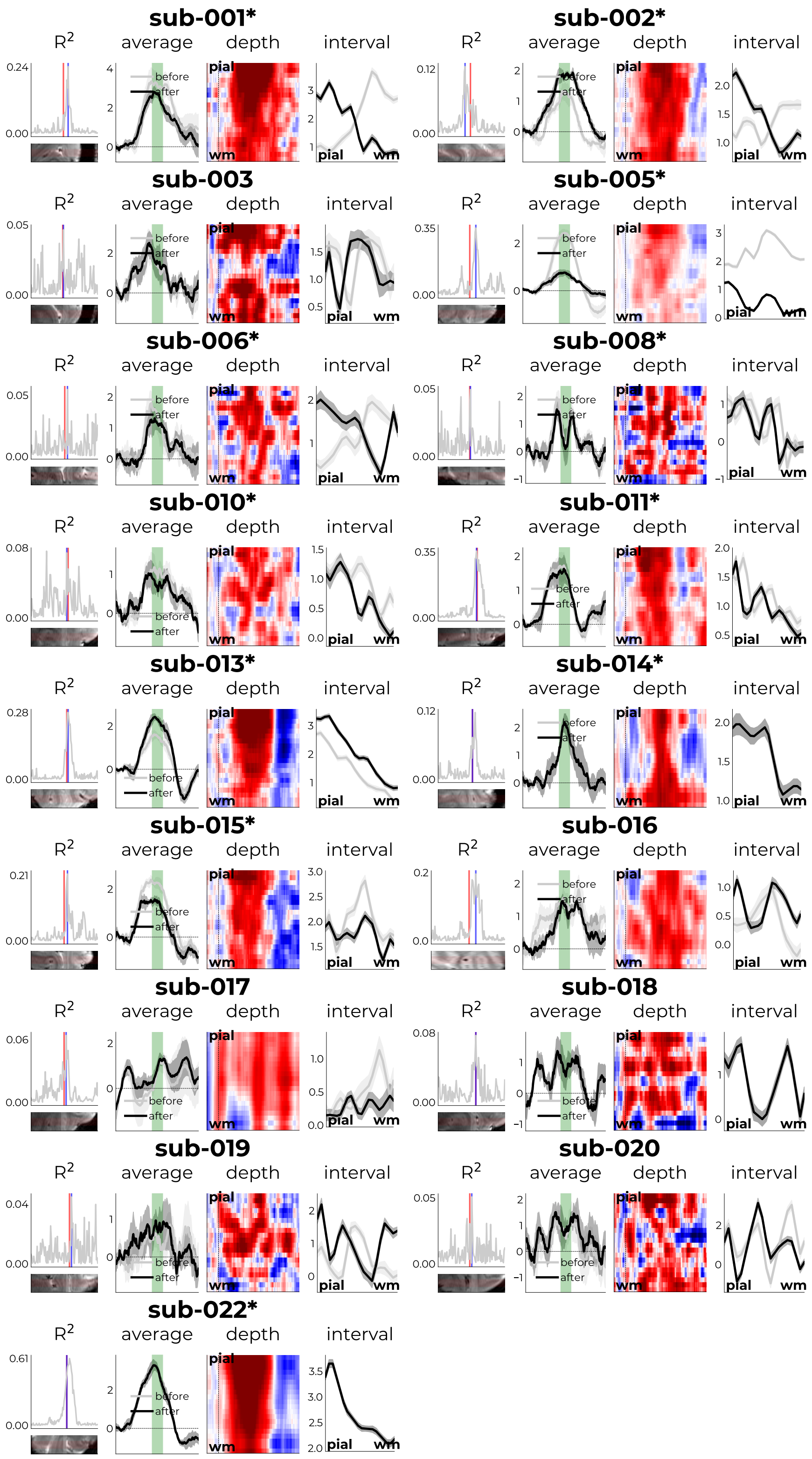

Figure S12: Voxel correction approach. First, a GLM model with the center event as reference was used to retrieve variance explained values across the line. Ideally, the variance explained should peak around the target coordinates (i.e., the middle of the line). To correct for small discrepancies between the anatomical voxel selection and functional acquisition (due to, e.g., motion), a correction was applied. Participants that passed both criteria are annotated with an asterisk (*). This figure is related to the section "*Quantification and statistical analysis*" of the STAR Methods.

References

1. Huber, L., Finn, E. S., Chai, Y., Goebel, R., Stirnberg, R., Stöcker, T., Marrett, S., Uludağ, K., Kim, S. G., Han, S. H., Bandettini, P. A., and Poser, B. A. (2021). Layer-dependent functional connectivity methods. Prog. Neurobiol. *207*, 101835.

2. Huber, L., Finn, E. S., Handwerker, D. A., Bönstrup, M., Glen, D. R., Kashyap, S., Ivanov, D., Petridou, N., Marrett, S., Goense, J., Poser, B. A., and Bandettini, P. A. (2020). Sub-millimeter fMRI reveals multiple topographical digit representations that form action maps in human motor cortex. NeuroImage *208*, 116463.

3. Huber, L., Handwerker, D. A., Jangraw, D. C., Chen, G., Hall, A., Stüber, C., Gonzalez-Castillo, J., Ivanov, D., Marrett, S., Guidi, M., Goense, J., Poser, B. A., and Bandettini, P. A. (2017). High-Resolution CBV-fMRI Allows Mapping of Laminar Activity and Connectivity of Cortical Input and Output in Human M1. Neuron *96*, 1253–1263.e7.

4. Huber, L., Tse, D. H. Y., Wiggins, C. J., Uludağ, K., Kashyap, S., Jangraw, D. C., Bandettini, P. A., Poser, B. A., and Ivanov, D. (2018). Ultra-high resolution blood volume fMRI and BOLD fMRI in humans at 9.4 T: Capabilities and challenges. NeuroImage *178*, 769–779.

5. Poser, B. A., Versluis, M. J., Hoogduin, J. M., and Norris, D. G. (2006). BOLD contrast sensitivity enhancement and artifact reduction with multiecho EPI: Parallel-acquired inhomogeneity-desensitized fMRI. Magn. Reson. Med. *55*, 1227–1235.

6. Raimondo, L., Priovoulos, N., Passarinho, C., Heij, J., Knapen, T., Dumoulin, S. O., Siero, J. C., and van der Zwaag, W. (2023). Robust high spatio-temporal line-scanning fMRI in humans at 7T using multi-echo readouts, denoising and prospective motion correction. J. Neurosci. Methods *384*, 109746.

7. Weber, B., Keller, A. L., Reichold, J., and Logothetis, N. K. (2008). The Microvascular System of the Striate and Extrastriate Visual Cortex of the Macaque. Cereb. Cortex *18*, 2318–2330.

8. Blinder, P., Tsai, P. S., Kaufhold, J. P., Knutsen, P. M., Suhl, H., and Kleinfeld, D. (2013). The cortical angiome: an interconnected vascular network with noncolumnar patterns of blood flow. Nat. Neurosci. *16*, 889–897.

9. Uludağ, K., and Blinder, P. (2018). Linking brain vascular physiology to hemodynamic response in ultra-high field MRI. NeuroImage *168*, 279–295.

10. de Hollander, G., van der Zwaag, W., Qian, C., Zhang, P., and Knapen, T. (2021). Ultra-high field fMRI reveals origins of feedforward and feedback activity within laminae of human ocular dominance columns. NeuroImage *228*, 117683.

11. Fracasso, A., Luijten, P. R., Dumoulin, S. O., and Petridou, N. (2018). Laminar imaging of positive and negative BOLD in human visual cortex at 7 T. NeuroImage *164*, 100–111.

12. Koopmans, P. J., Barth, M., Orzada, S., and Norris, D. G. (2011). Multi-echo fMRI of the cortical laminae in humans at 7T. NeuroImage *56*, 1276–1285.

13. Polimeni, J. R., Fischl, B., Greve, D. N., and Wald, L. L. (2010). Laminar analysis of 7T BOLD using an imposed spatial activation pattern in human V1. NeuroImage *52*, 1334–1346.

14. Siero, J. C. W., Petridou, N., Hoogduin, H., Luijten, P. R., and Ramsey, N. F. (2011). Cortical depth-dependent temporal dynamics of the BOLD response in the human brain. J. Cereb. Blood Flow Metab. *31*, 1999–2008.

15. van der Zwaag, W., Francis, S., Head, K., Peters, A., Gowland, P., Morris, P., and Bowtell, R. (2009). fMRI at 1.5, 3 and 7 T: Characterising BOLD signal changes. NeuroImage *47*, 1425–1434.

16. van Dijk, J. A., Fracasso, A., Petridou, N., and Dumoulin, S. O. (2020). Linear systems analysis for laminar fMRI: Evaluating BOLD amplitude scaling for luminance contrast manipulations. Sci. Rep. *10*, 5462.

17. Heij, J., Raimondo, L., Siero, J. C. W., Dumoulin, S. O., van der Zwaag, W., and Knapen, T. (2023). A selection and targeting framework of cortical locations for line-scanning fMRI. Hum. Brain Mapp. *44*, 5471–5484.

18. Markuerkiaga, I., Barth, M., and Norris, D. G. (2016). A cortical vascular model for examining the specificity of the laminar BOLD signal. NeuroImage *132*, 491–498.

19. Marquardt, I., Schneider, M., Gulban, O. F., Ivanov, D., and Uludağ, K. (2018). Cortical depth profiles of luminance contrast responses in human V1 and V2 using 7 T fMRI. Hum. Brain Mapp. *39*, 2812–2827.

20. Boas, D. A., Jones, S. R., Devor, A., Huppert, T. J., and Dale, A. M. (2008). A vascular anatomical network model of the spatio-temporal response to brain activation. NeuroImage *40*, 1116–1129.

21. Uludağ, K., Müller-Bierl, B., and Uğurbil, K. (2009). An integrative model for neuronal activity-induced signal changes for gradient and spin echo functional imaging. NeuroImage *48*, 150–165.

22. Hubel, D. H., and Wiesel, T. N. (1972). Laminar and columnar distribution of geniculo-cortical fibers in the macaque monkey. J. Comp. Neurol. *146*, 421–450.

23. Felleman, D. J., and van Essen, D. C. (1991). Distributed hierarchical processing in the primate cerebral cortex. Cereb. Cortex *1*, 1–47.

24. Bijanzadeh, M., Nurminen, L., Merlin, S., Clark, A. M., and Angelucci, A. (2018). Distinct Laminar Processing of Local and Global Context in Primate Primary Visual Cortex. Neuron *100*, 259–274.e4.
